# Long-Term Unemployment Is Associated with Short Telomeres in 31-Year-Old Men: An Observational Study in the Northern Finland Birth Cohort 1966

**DOI:** 10.1371/journal.pone.0080094

**Published:** 2013-11-20

**Authors:** Leena Ala-Mursula, Jessica L. Buxton, Ellen Ek, Markku Koiranen, Anja Taanila, Alexandra I. F. Blakemore, Marjo-Riitta Järvelin

**Affiliations:** 1 Institute of Health Sciences, University of Oulu, Oulu, Finland; 2 Section of Investigative Medicine, Division of Diabetes, Endocrinology and Metabolism, Imperial College, London, United Kingdom; 3 Unit of Primary Care, Oulu University Hospital, Oulu, Finland; 4 Department of Epidemiology and Biostatistics, MRC Health Protection Agency Centre for Environment and Health, School of Public Health, Imperial College London, London, United Kingdom; 5 Biocenter Oulu, University of Oulu, Oulu, Finland; 6 Department of Children and Young People and Families, National Institute for Health and Welfare, Oulu, Finland; Virginia Commonwealth University, United States of America

## Abstract

**Objective:**

Life stress resulting from early-life experiences and domestic stress is linked with shorter leukocyte telomere length (LTL), but evidence on employment-related stress is scarce. We explored whether unemployment in early adulthood is associated with shorter LTL, a potential biomarker of premature aging.

**Methods:**

We used data from 5620 men and women belonging to the Northern Finland Birth Cohort 1966. Individually registered unemployment days in 1995–97 were compared with data on biological, behavioral and socioeconomic health predictors and existing medical conditions obtained by surveys and clinical examinations at follow-up in 1997–98. Mean LTL at follow-up was measured by multiplex quantitative real-time PCR. We calculated odds ratios and their 95% confidence intervals (CI) of belonging to the sex-stratified shortest decile of standardized relative mean LTL according to the categories of: 0, <260, <500 and over 500 unemployment days, representing 0, <1, <2 and over 2 calendar years.

**Results:**

Among men, unemployment exceeding 500 days during three years was associated with having shorter LTL at follow-up, compared to being continuously employed. The corresponding odds ratio was 2.61 (95% CI 1.16 to 5.85) in the fully adjusted model. Such an association was not found among women in this study.

**Conclusions:**

Long-term unemployment in early adulthood is associated with shorter LTL among men.

## Introduction

Telomeres are DNA-protein complexes that cap and protect linear chromosomes from degradation and fusion. Telomeres shorten in cells of proliferative tissues after each division, with attrition rates exacerbated by oxidative stress and inflammation, and attenuated by the enzyme telomerase. There is wide inter-individual variation in telomere length and its trajectories during the life course. [1], [2], [3], [4], [5] When a critically short telomere length is reached, the cell enters senescence or undergoes programmed cell death. Leukocyte telomere length (LTL) has been proposed as a biomarker of biological aging [6], although this remains to be confirmed through further longitudinal studies [3], [4]. Shorter LTL is associated with various environmental and behavioral determinants of ill health and predicts the onset of age-related diseases and earlier death [7], [8], [9], [10], [11]. Accelerated telomere attrition has also been suggested as a potential mechanism underlying the health adversities emerging from life stress. Specifically, shorter LTL has been associated with chronic care-giving stress and early-life adversities in a dose-response manner [12], [13]. However, less is known about the association between LTL and stress related to working life.

In a US study, long-term full-time work and the holding of multiple jobs were associated with shorter LTL among 608 women aged 35–74 years [14]. Participants with minimal work histories (<25% of potential work life span), though excluded from further analyses, also had shorter telomeres. In another US study of 981 men and women aged between 45–84 years, mean telomere length did not differ across employment status, occupational or job strain category [15]. A population-based study in Finland revealed an association between work-related exhaustion and shorter LTL among 2911 men and women aged 30–64 years [16]. A comparative study of different life stressors and LTL was conducted in a UK population-based sample of 4441 women aged 41–80 years using a social adversity exposure history instrument; no association was found with the work-related events measured, such as retirement, redundancy or being fired [17]. Indirectly, employment has been studied through occupation-based measures of socioeconomic status in relation to LTL. Among UK female twins [18], short telomeres were associated with low occupational level, but this finding has not been replicated in other studies [19], [20], [21], [22]. In a population-based case-control study of 1542 Scottish men aged 45–64 years, the only occupation-based survey measure associated with shorter LTL was being out of work [20].

From a public health perspective, unemployment is a widespread [23] stressor related to working life. Increasing evidence is linking unemployment with poor health across various outcomes, including mortality [24]. However, evidence for the association of unemployment with telomere length, a potential biomarker of cellular aging, is lacking. Furthermore, the participants in most previous studies of employment-related exposures and LTL span a wide age-range. This is a limitation not only because of the age-related decline in LTL [25], but also because rapid changes in working life patterns produce very different circumstances for different generations.

In this study on the Northern Finland Birth Cohort 1966 (NFBC1966), we aimed to explore how the participants' registered unemployment days during a three-year period associate with shortness of LTL at the end of the follow-up, at 31 years of age. The large sample enabled us to study generation-level working life experiences together with a wide range of potential confounders. Our data showed an association between long-term unemployment and shorter LTL among men in their early adulthood.

## Methods

### Participants

The data are from the NFBC1966 study, based on 12 058 live-born children in Northern Finland (96 percent of all births, 6169 boys and 5889 girls) [26], [27]. The database started with pregnancies in 1965 and comprises questionnaire data, clinical examinations, hospital records and national register data.

The latest completed follow-up was conducted in 1997 at the age of 31 years: a questionnaire was posted to all 11 541 alive and reachable cohort members, of which 8690 (75.3%) answered. The 8463 cohort members living in Northern Finland or in the Helsinki area were invited to a clinical examination in 1997/1998 and 6033 (71.3%) participated. LTL measurements were obtained for the participants in the clinical examinations who both gave consent (n = 6007) and for whom DNA from banked blood samples drawn in 1997/1998 were available (n = 5753, 95.4% of those clinically examined). The final sample of this study consisted of 5620 participants (97.7% of those with DNA sample, 93.6% of those with clinical data), the 2713 men (48%) and the 2907 women (52%) from which laboratory data on LTL was successfully obtained.

Previous studies of sample attrition found that between 1966 and 1982 the dropouts were slightly more often men than women and that by 1997 the subjects with psychiatric disorders were slightly underrepresented compared to those without psychiatric disorders [26], [27], [28].

### Ethics statement

The Ethics Committee of the Northern Ostrobothnia Hospital District approved the study. All participants gave written informed consents to use their data for research.

### Leukocyte telomere length (LTL) measurements

Leukocyte telomere length (LTL) was measured in genomic DNA samples prepared from the banked blood specimens using a multiplex quantitative real-time PCR method [29] with minor modifications as described in detail previously [30]. All PCRs were carried out in white 384-well plates on a CFX384 Real-time PCR detection system (Bio-Rad). Five serial dilutions of a reference sample (leukocyte DNA from a 42-year-old non-cohort female) spanning 5–50 ng were run in triplicate on each plate, in addition to a no-template control (NTC). Human beta-globin (Hgb) was used as the single copy reference gene.

Following amplification and data collection, the CFX manager software (Bio-rad) was used to generate standard curves for the reference DNA dilutions, one for the telomere signal (T) and one for the single copy gene signal (S). Telomere measurements for each sample were calculated as T/S ratios, a relative measure of the amplification of the telomeric DNA sequence compared to that of the single copy gene. The overall mean coefficient of variation (CV) for T/S values of duplicate test samples on the same plate was 5%, and the mean inter-run CV for selected samples was 6.2%. Samples with extreme T/S values (>3 standard deviations from the population mean) and those with a CV>15% after repeat measurement were excluded from the analysis.

To control for batch variation between the measurements from the 42 different plates, the T/S values of mean length were first log transformed, then standardized by plate separately for the men and women [31], measuring the standardized relative mean telomere length. With stratification for sex, the known sex-dependence of telomere length and attrition [25] was taken into account. As the cells enter senescence after telomeres reach critically short length, the metrics of the shortest telomeres are of particular interest biologically [4]. Since the qPCR method cannot be used to obtain absolute telomere length, the potential biological relevance of short LTL was investigated by focusing on the group of participants belonging to the sex-stratified shortest decile of standardized relative mean LTL, indicating prevalence of telomere shortness across each potential predictor.

### Unemployment measurements

The individually recorded data on unemployment days were derived from the registers of the Finnish National Social Insurance Institute. These registers are close to comprehensively kept, as they serve as the basis for determining the duration of unemployment and thus the eligibility for unemployment allowance.

The unemployment days indicate the days when the person is fit and available for work but not employed or studying. The person is entitled to earnings-based or basic unemployment allowance for up to 500 days, which is the cumulative maximum with or without interruptions. Each day in unemployment must be confirmed by personal signature in the employment agency. Thus, the days employed or self-employed and the periods when the person is not available for work, such as family or sick leave and full-time studying, are reliably excluded from the unemployment figures. Register data on unemployment was available for 5578 participants, 99.3 percent of those whose telomere length was measured.

To assess the association of unemployment of varying duration with future LTL, we obtained from the registers the unemployment data in 1995–97, from three years before the blood samples used for DNA preparations were taken in 1997–98. As the unemployment allowance is paid for 5 days per week for 52 weeks per year, resulting in 260 days per year, the range for potential exposure to unemployment was 0–780 days during the three-year follow-up.

The categories of cumulative unemployment in 1995–97 were determined as 0 days = no unemployment, 1–260 days = unemployment lasting less than one calendar-equal year altogether, 261–500 days = unemployment for more than one calendar-equal year, and over 500 days = long-term unemployment, equal to more than two years of unemployment during the three-year follow-up and exceeding the 500 days' time limit over which the person is no longer entitled to the unemployment allowance and is only eligible for the smaller labor market subsidy.

### Covariates

#### Early biological factors

*Paternal age at birth*, previously [32] associated with longer telomeres, was obtained from the registers. *Birth weight* reflecting the early status of health of the participant was recorded from the hospital files [26].

#### Social functioning

Since having fewer *years of education* has been associated with shorter telomeres in middle and old age [21], we studied the impact of educational status at 31 years by using institution-based registered information on years in education from Statistics Finland, categorized into three levels: basic (<10 years), secondary (10–12 years) and tertiary (>12 years) education. The respondents' *socioeconomic status* was obtained from the self-reported level of occupation according to Statistics Finland [33] and categorized as follows: upper white collar, lower white collar, blue collar, farmer and other (such as entrepreneur, student, home-maker, pensioner etc). *Marital status* was derived from the survey and dichotomized as married/cohabiting vs. single/divorced/widowed. *Family status* was determined using survey information of having biological children, and dichotomized as yes vs. no.

#### Behavioral and biological health risks

*Body mass index* (BMI) was calculated from weight and height measurements during the clinical examination and categorized as <25, 25–30 and >30 kg/m^2^. *Smoking* was categorized from the self-reports as no, 1–10 and over 10 cigarettes smoked per day. *Use of alcohol* was measured by self-reports of the average consumption during the previous year, detailing the frequency and the usual amount of each type of beverage (beer, wine, spirits) per drinking occasion. The average amount of alcohol consumed daily was calculated and categorized into three levels: 0, <40, >40 grams per day for men and as 0, <20, >20 grams per day for women, to indicate consumption levels of none/light/risk-inducing, respectively. The subjects were classified into four groups for *leisure-time physical activity* (very active, active, moderately active and inactive) according to the frequency, intensity and duration of physical activity they had been engaged in [34].

#### Common medical conditions

Existing doctor-diagnosed illnesses and conditions were derived from the survey question: Have you ever been diagnosed by a doctor as having diabetes, hypertension, heart insufficiency or angina pectoris to indicate *somatic illness* (yes/no) and psychosis or depression to indicate *psychiatric illness* (yes/no).

### Statistical analyses

All analyses were conducted separately for men and women, using SAS software version 9.2. Questionnaire and registered data were used for all 5620 participants with telomeres measured, variables treated as missing if necessary.

To depict the crude association, we first drew scatterplots of standardized relative mean LTL by the continuous number of days unemployed in 1995–97 and explored the association by fitting a cubic function. The goodness of fit of the cubic function was estimated by F-statistics in analysis of variance. Percentages of the participants with the shortest telomeres, i.e. of those belonging to the shortest decile of standardized relative mean LTL were then quantified in each category of unemployment and covariates, and the corresponding prevalence ratios together with their 95% confidence intervals (CI) relative to the reference categories were calculated.

We conducted logistic regression analyses to present crude odds ratios (OR) and their 95% CI of belonging to the shortest decile of standardized relative mean LTL by the categories of unemployment exposure, then further adjusted for the potential predictors, grouped in categories of early biology, social functioning, behavioural and biological health risks and common medical conditions, and lastly, adjusted for all the aforementioned. Chi-square tests were used to further analyse the significance of the OR estimates.

## Results

**Figure 1:**
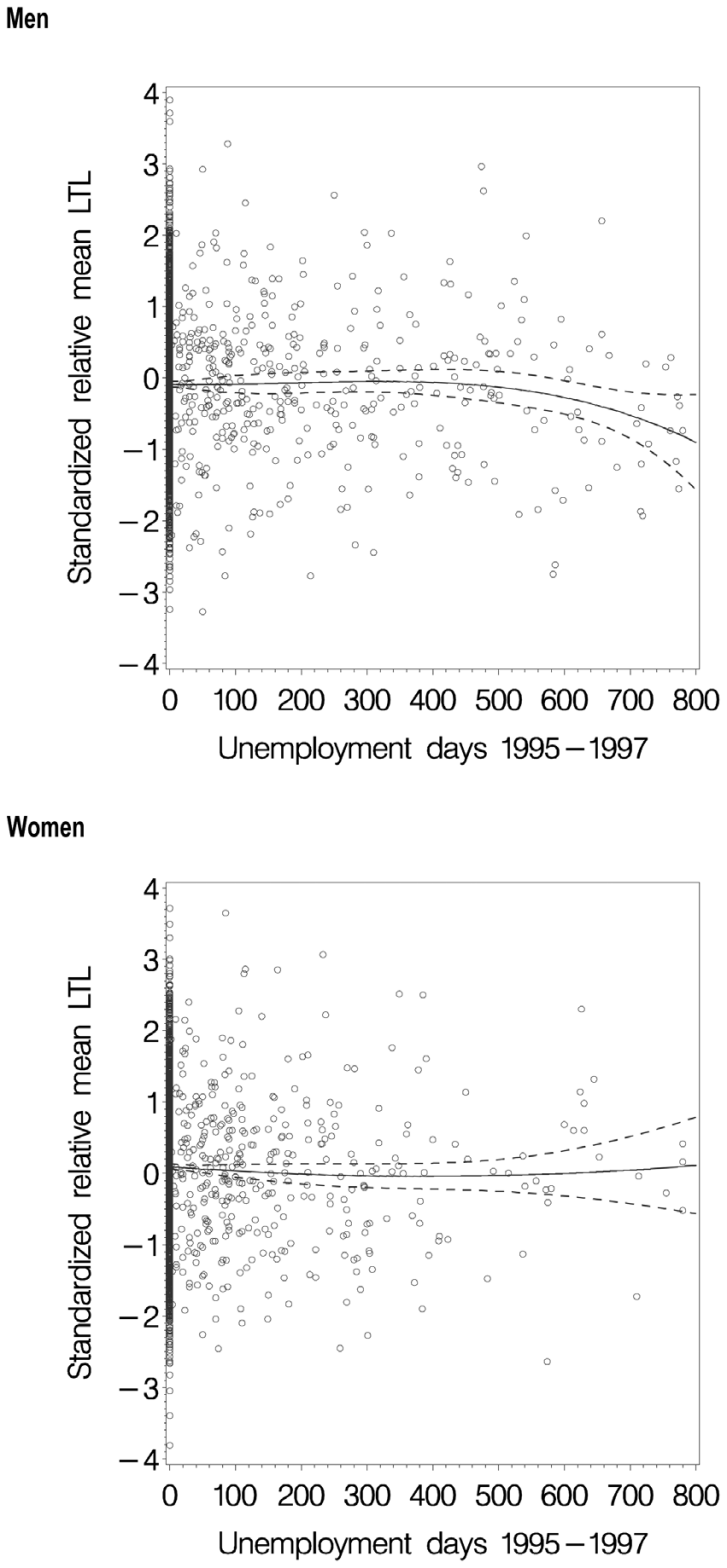
Scatterplots of LTL (standardized relative mean leukocyte telomere length) at follow-up in relation to the number of unemployment days in the preceding three years 1995–97. The lines indicate the LTL curves fitted to cubic function of unemployment days and the dashed lines their 95% confidence intervals.

LTL positively associated with female sex: the mean log-transformed T/S value was 0.11 for men and 0.16 for women (*P* for difference <0.001). The distribution of unemployment days was skewed, ranging from 0 days (median) to the maximal 780 days for both sexes. Higher exposure to unemployment days associated with male sex: during the three years, the mean number of unemployment days was 34 days (SD 112) for the men and 22 days (SD 80) for the women. As illustrated in the scatterplots of the mean LTL against unemployment days in 1995–97 (figure 1), an association between a long-term exposure to unemployment and shorter mean LTL was found among the men (*P* = 0.026 among men, *P* = 0.880 among women). Also the proportion of those belonging to the sex-stratified shortest decile of LTL was heightened among the men exposed to over 500 days of unemployment, as shown in table 1. Regarding the potential confounders, the risk of LTL belonging to the shortest decile (table 1) was also associated with not having children among the men, and having a father aged under 21 at birth among the women.

**Table 1:** Frequencies, percentages and prevalence ratios (with 95% confidence intervals, CI) of the participants belonging to the shortest decile of standardized relative mean leukocyte telomere length (LTL) at follow-up in relation to the categories of biological, socioeconomic, behavioural and medical covariates and exposure to unemployment days in the preceding three years.

			MEN				WOMEN			
Entity	Exposure	Category		Short LTL		Prevalence ratio and 95% CI		Short LTL		Prevalence ratio and 95% CI
			n	n	%		n	n	%	
**a. Early biology**	**Paternal age at birth**	21–40 y (ref)	2241	217	9.7	1.00	2396	248	10.4	1.00
		<21 y	72	11	15.3	1.58 (0.90 to 2.76)	81	16	19.8	1.91 (1.21 to 3.01)
		>40 y	323	35	10.8	1.12 (0.80 to 1.57)	334	23	6.9	0.67 (0.44 to 1.00)
	**Birth weight**	2500–4000 g(ref)	2142	226	10.6	1.00	2511	256	10.2	1.00
		<2500 g	76	6	7.9	0.75 (0.34 to 1.63)	92	15	16.3	1.60 (0.99 to 2.58)
		>4000 g	495	40	8.1	0.77 (0.56 to 1.06)	304	25	8.2	0.81 (0.54 to 1.20)
**b. Social functioning**	**Education**	Tertiary (>12 y, ref)	692	67	9.7	1.00	778	86	11.1	1.00
		Secondary (10–12 y)	1687	167	9.9	1.02 (0.78 to 1.34)	1913	190	9.9	0.90 (0.71 to 1.14)
		Basic (>10 y)	334	38	11.4	1.18 (0.81 to 1.71)	216	20	9.3	0.84 (0.53 to 1.33)
	**SES**	Upper white collar (ref)	526	52	9.9	1.00	511	59	11.5	1.00
		Lower white collar	526	51	9.7	0.98 (0.68 to 1.42)	1429	154	10.8	0.93 (0.70 to 1.24)
		Blue collar	1225	137	11.2	1.13 (0.84 to 1.53)	568	46	8.1	0.70 (0.49 to 1.01)
		Farmer	133	6	4.5	0.46 (0.20 to 1.04)	82	4	4.9	0.42 (0.16 to 1.13)
		Other	232	22	9.5	0.96 (0.60 to 1.54)	246	28	11.4	0.99 (0.65 to 1.51)
	**Marital status**	Married/cohabiting (ref)	1839	178	9.7	1.00	2210	217	9.8	1.00
		Other	842	90	10.7	1.10 (0.87 to 1.40)	674	76	11.3	1.15 (0.90 to 1.47)
	**Family status**	Child(ren) (ref)	1415	127	9.0	1.00	2009	203	10.1	1.00
		No child	1235	139	11.3	1.25 (1.00 to 1.58)	865	88	10.2	1.01 (0.79 to 1.28)
**c. Behavioural health risks**	**BMI**	25–30 (ref)	1096	113	10.3	1.00	660	76	11.5	1.00
		<25	1370	132	9.6	0.93 (0.74 to 1.19)	1952	189	9.7	0.84 (0.65 to 1.08)
		>30	227	25	11.0	1.07 (0.71 to 1.61)	273	29	10.6	0.92 (0.62 to 1.38)
	**Alcohol consumption**	no (ref)	285	21	7.4	1.00	595	72	12.1	1.00
		1–40(m)1–20(w) g/day	2179	227	10.4	1.41 (0.92 to 2.17)	2094	202	9.6	0.80 (0.62 to 1.03)
		>40(m)>20(w) g/day	159	13	8.2	1.11 (0.57 to 2.16)	129	14	10.9	0.90 (0.52 to 1.54)
	**Smoking**	no (ref)	1361	125	9.2	1.00	1785	181	10.1	1.00
		1–10 cigarettes	485	47	9.7	1.06 (0.77 to 1.45)	727	78	10.7	1.06 (0.82 to 1.36)
		>10 cigarettes	797	93	11.7	1.27 (0.99 to 1.64)	347	33	9.5	0.94 (0.66 to 1.33)
	**Leisure-time physical activity**	very active (ref)	814	85	10.4	1.00	1186	119	10.0	1.00
		active	875	79	9.0	0.86 (0.65 to 1.16)	920	97	10.5	1.05 (0.82 to 1.35)
		moderately active	362	34	9.4	0.90 (0.62 to 1.31)	397	42	10.6	1.05 (0.76 to 1.47)
		inactive	627	72	11.5	1.10 (0.82 to 1.48)	381	35	9.2	0.92 (0.64 to 1.31)
**d. Medical conditions**	**Somatic**	No(ref)	2339	231	9.9	1.00	2450	246	10.0	1.00
		Yes	374	41	11	1.11 (0.81 to 1.52)	457	50	10.9	1.09 (0.82 to 1.45)
	**Psychiatric**	No(ref)	2617	264	10.1	1.00	2756	278	10.1	1.00
		Yes	96	8	8.3	0.83 (0.42 to 1.62)	151	18	11.9	1.18 (0.76 to 1.85)
**e. Unemployment**	**Days (5 per week) in previous three years**	0 (ref)	2276	223	9.8	1.00	2510	251	10.0	1.00
		1–260	277	28	10.1	1.03 (0.71 to 1.50)	295	31	10.5	1.05 (0.74 to 1.50)
		261–500	90	10	11.1	1.13 (0.62 to 2.06)	59	8	13.6	1.36 (0.70 to 2.61)
		>500	48	10	20.8	2.13 (1.21 to 3.74)	23	2	8.7	0.87 (0.23 to 3.29)
**f. Total**			2713	272	10.0		2907	296	10.2	

The main result is presented in table 2. Compared to the men who were continuously employed, the men exposed to unemployment for more than 500 days during the preceding three years had a 2.4 –fold OR of belonging to the shortest decile of telomere length. The result was not attenuated by adjusting for the grouped potential covariates, not even after taking into account medical conditions. In the fully adjusted model, the OR of having short telomeres was 2.6 –fold among the long-term unemployed men compared to those who were continuously employed (*P* = 0.020). There were fewer women exposed to over 500 days or even over 365 days of employment, and no association between unemployment exposure categories and LTL in women was found in this study.

**Table 2:** Odds ratios (OR) and their 95% confidence intervals (CI) of belonging to the shortest decile of standardized relative mean leukocyte telomere length (LTL) at the end of follow-up by exposure to unemployment days during three years, unadjusted and adjusted for potential confounders presented in table 1.

		OR (95%CI) of LTL shortness (in the shortest decile) according to the logistic regression model												
		Unadjusted		Adjusted for early biology*		Adjusted for social functioning**		Adjusted for behavioral health risks***		Adjusted for medical conditions****		Fully adjusted*****		
	Un-employment days in 1995–97	OR	95% CI	OR	95% CI	OR	95% CI	OR	95% CI	OR	95% CI	OR	95% CI	*P* for OR estimate
**Men**	0 (ref)													
	1–260	1.04	(0.68 to 1.57)	0.99	(0.64 to 1.52)	0.97	(0.63 to 1.50)	0.98	(0.64 to 1.51)	1.05	(0.69 to 1.60)	0.92	(0.58 to 1.45)	0.706
	261–500	1.15	(0.59 to 2.25)	1.17	(0.60 to 2.30)	0.95	(0.46 to 1.94)	1.10	(0.54 to 2.24)	1.19	(0.61 to 2.34)	0.97	(0.45 to 2.08)	0.936
	>500	2.42	(1.19 to 4.93)	2.46	(1.20 to 5.02)	2.38	(1.13 to 5.00)	2.50	(1.17 to 5.34)	2.63	(1.27 to 5.44)	2.61	(1.16 to 5.85)	0.020
**Women**	0 (ref)													
	1–260	1.06	(0.71 to 1.57)	1.00	(0.66 to 1.50)	1.14	(0.76 to 1.71)	1.00	(0.66 to 1.52)	1.05	(0.71 to 1.55)	1.00	(0.65 to 1.56)	0.986
	261–500	1.41	(0.66 to 3.01)	1.50	(0.70 to 3.22)	1.77	(0.81 to 3.84)	1.46	(0.68 to 3.15)	1.40	(0.66 to 2.98)	1.85	(0.83 to 4.11)	0.131
	>500	0.86	(0.20 to 3.68)	0.97	(0.22 to 4.20)	1.12	(0.26 to 4.91)	0.94	(0.22 to 4.05)	0.83	(0.19 to 3.58)	1.49	(0.33 to 6.71)	0.600

* early biology: paternal age at birth, birthweight.

** social functioning: years of education at 31 years, occupation-based socioeconomic status at 31 years, marital status, family status.

*** biological and behavioral health risks: BMI, smoking, consumption of alcohol, leisure-time physical activity.

**** medical conditions: self-reported history of doctor-diagnosed somatic (diabetes, hypertension, heart insufficiency or angina pectoris)or psychiatric (psychosis, depression) illness.

***** all aforementioned.

## Discussion

Among 31-year-old men, unemployment exceeding 500 days or two calendar years within the preceding three years was associated with shorter LTL, even after adjusting for potential biological, behavioral, socioeconomic and medical confounders. The stress resulting from long-term unemployment appeared to be of key importance, as no dose-response relation regarding lower levels of exposure emerged. Unemployment has been linked with numerous poor health outcomes including mortality [24], and now also with shorter telomere length, a potential biomarker of premature aging.

A strength of the design was the triangulation of registered data on unemployment with individual measurements of telomere length, thus avoiding memory and reporting bias on exposure. The large, ethnically homogenous [35] population-level sample covering both sexes and all levels of socioeconomic status, and without intergenerational differences in working life circumstances or conventions also enabled us to control for a large set of potential confounders. The robustness of the result suggests that we not merely identified those individuals with long-standing poor health or risky lifestyle. A limitation is the cross-sectional LTL measurement, so individual trajectories of telomere length could not be studied [5]. Also, unmeasured working life or health issues may underlie the results. Our analyses were restricted to leukocyte DNA samples. Within individuals however, lengths and shortening rates of telomeres have been shown to strongly correlate between tissues [36]. The measurement of telomeres was conducted using a well-established qPCR technique enabling analyses of large numbers of samples, with low levels of inter-assay variability. The reliability of the LTL data obtained is further evidenced by the fact that it shows the expected sex difference, and that it has been used to identify robust genetic associations for this trait [37].

To our knowledge, this is the first study to directly address the relationship between unemployment and telomere length. The few studies on work-related factors that have included unemployment as one potential predictor of telomere length have not found clear associations [14], [15]. However, there have either been too few unemployed participants to be included in the further analyses or the participants on-leave and unemployed have been treated as one group in the analyses. Considering the dependence of telomere length on age and ethnicity, the heterogeneity of the samples in the previous studies may have made it difficult to identify associations with unemployment. In addition, cross-sectional classifications are problematic in differentiating temporary unemployment from long-term worklessness.

In contrast to the previously observed dose-response association between early-life and domestic stressors and shorter LTL [12], [13], it was only after an excessive exposure of over 500 unemployment days that the odds of short telomeres grew higher. In our sample, notably fewer women than men were exposed to unemployment totaling more than 500 days. This reflects the sex-specific family leave figures for the stage of life when children are born, providing women with more diverse options for socioeconomic activity, and possibly reducing vulnerability to disappointments in any one area of life. The traditional view of the man as the wage earner and the woman as the home maker may also mean that unemployment is more harmful to men than to women. However, attitudes towards working life are changing, and the picture is probably more diverse among both sexes. For decades, it has been the norm for Finnish women to work full-time, at least later in life after the busy years of building a family have passed [38]. It remains a possibility that prolonged unemployment is harmful for both sexes, at least when involuntary.

The life stage of our participants raises public health concerns about the long-term effects of worklessness in early adulthood, especially considering the internationally high rates of juvenile unemployment. Further studies of telomere dynamics across life course trajectories are needed to estimate the predictive value of our finding to population-level morbidity [4], with regard to the previous studies showing that those with shorter telomeres are at increased risk of diseases and dying early [10], [11]. Unemployment among young adults has previously been associated with premature mortality [39]. Sex-specific patterns were found in a Swedish follow-up study [40]; prolonged unemployment predicted mortality with a cubic function among the men and linearly for the women. Recently, an influential review [41] highlighted worklessness as a major cause of ill health, mortality, health inequality, deprivation and child poverty. The message was clear: being in work is generally better for health and well-being than being out of work. Steps have since been taken to direct focus on employment as an essential part of general health promotion. In practical terms, avoiding unnecessary sickness absence and better clinical accuracy in assessing work ability are needed, to minimize the patients' risk of becoming unemployed [41], [42], [43].

To conclude, our results add to the literature connecting unemployment with poor health. Prolonged unemployment among men in their early adulthood is associated with shorter LTL, a potential indicator of accelerated biological ageing. This novel cross-sectional finding warrants replication in other settings, subgroups and stages of life, extending measurements to all aspects of socioeconomic activity. Given the high individual variability in LTL and dynamics of attrition rates, multiple measurements of LTL and predictors are needed to determine the mechanisms underlying this finding.
